# fNIRS as a biomarker for X-linked neurodevelopmental disorders: leveraging visual processing to assess brain function?

**DOI:** 10.3389/fnins.2025.1560786

**Published:** 2025-05-07

**Authors:** Elena Scaffei, Chiara Bosetti, Roberta Battini, Michela Fagiolini, Laura Baroncelli

**Affiliations:** ^1^Department of Developmental Neuroscience, IRCCS Stella Maris Foundation, Pisa, Italy; ^2^Department of Clinical and Experimental Medicine, University of Pisa, Pisa, Italy; ^3^National Research Council (CNR), Institute of Neuroscience, Pisa, Italy; ^4^Department of Neurology, Boston Children's Hospital, F. M. Kirby Neurobiology Center, Harvard Medical School, Boston, MA, United States

**Keywords:** fNIRS, visual stimulation, brain biomarker, neurodevelopmental disorders, X-linked rare disease

## Abstract

Neurodevelopmental disorders (NDDs) cause profound intellectual and physical impairment, yet therapeutic progress remains hindered by the lack of quantitative, unbiased, and non-invasive biomarkers to monitor disease onset and progression. Visual evoked potentials (VEPs) have emerged as promising functional biomarkers for X-linked NDDs, with reduced VEP amplitudes correlating with disease severity. Complementary approaches, like functional Near Infrared Spectroscopy (fNIRS), offer a non-invasive additional tool to assess brain metabolism, monitor disease progression, and evaluate therapeutic responses. This perspective explores the potential of fNIRS in studying visually evoked hemodynamic responses (vHDR) across different age groups, demonstrating its reliability in capturing task-specific cortical activation and tracking brain maturation even in challenging populations. Notably, fNIRS identifies unique vHDR patterns in conditions like optic neuritis, myopia, glaucoma, and migraines, validating its role as a biomarker for disease severity and treatment efficacy. Moreover, fNIRS has proven effective in detecting early neural deficits in high-risk infants, including pre-term newborns. Preclinical studies support that visually induced hemodynamic changes can differentiate healthy from pathological conditions in X-linked NDDs. However, direct evidence from human cohorts with X-linked NDDs remains limited, highlighting the urgent need for further research to validate the potential of visual fNIRS as a reliable functional biomarker in clinical settings. To enhance clinical relevance, the development of standardized protocols, engaging stimuli, and age-stratified analyses is also crucial for improving diagnostic accuracy, tracking neurodevelopmental trajectories, and evaluating therapeutic interventions.

## 1 Introduction

Neurodevelopmental disorders (NDDs) pose a significant global public health challenge, impacting millions of children and their families across the world. These chronic and heterogeneous conditions are often underdiagnosed, with an estimated prevalence exceeding 1.5% of the global population (Chung et al., 2022). Over 90 genes on the X chromosome have been linked to NDDs, which encompass rare disorders characterized by severe intellectual and physical disabilities (Lumsden and Urv, 2023). Despite the urgent need for effective treatments, progress in therapeutic development remains constrained by the lack of robust translational endpoints and reliable, non-invasive biomarkers essential for accelerating preclinical and clinical testing (Kearns and Artman, 2015; Budimirovic et al., 2017; Krainc et al., 2023).

Recent animal model studies have demonstrated that the visual system serves as a powerful proxy for assessing overall brain function (Durand et al., 2012; Della Sala and Pizzorusso, 2014; Mazziotti et al., 2020). Visual evoked potential (VEP) electrophysiological recordings have revealed significantly reduced VEP amplitudes and diminished visual acuity in *Mecp2* mutant mice compared to their wild-type (WT) littermates. Notably, the reduction in VEP amplitude is most pronounced in animals with greater phenotypic severity and more advanced RTT-like symptoms, suggesting that VEP amplitude reflects overall neurological health and disorder progression (LeBlanc et al., 2015). Importantly, similar findings have been reported in RTT patients, who exhibit significantly diminished P1 amplitudes, which worsen as the disorder advances (Stauder et al., 2006; LeBlanc et al., 2015; Saby et al., 2021). These data underscore the potential clinical utility of P1 amplitude as a translatable biomarker. Furthermore, recent analyses have extended these observations to other X-linked disorders, with similar results replicated in both animal models and patients with CDKL5 Deficiency (Mazziotti et al., 2017; Saby et al., 2022), and MECP2 duplication syndrome (Saby et al., 2023).

These findings highlight neurophysiological measures, such as VEPs, as valuable, quantitative, and unbiased biomarkers for assessing disease severity and progression in X-linked NDDs. Their potential for repeated, non-invasive longitudinal assessments also makes them highly suitable for clinical trial readiness (LeBlanc et al., 2015; McPartland, 2016; Sidorov et al., 2017). However, the development of additional biomarkers is critical for advancing our understanding of these disorders and refine therapeutic strategies. Given that disruptions in energy metabolism are a key pathological mechanism in many X-linked disorders (Ghirardini et al., 2021; Oyarzábal et al., 2021; van Bergen et al., 2021), assessing cerebral blood flow and oxygen consumption offers a complementary and highly sensitive approach for quantifying functional changes in neural circuits. This strategy provides crucial insights into neurovascular coupling, enhancing our ability to monitor disease progression and therapeutic efficacy.

The functional Near Infrared Spectroscopy (fNIRS) is a non-invasive optical imaging technique that has gained increasing recognition for analyzing energy metabolism in the brain (Strangman et al., 2002; Cui et al., 2011; Chen et al., 2020). By detecting hemodynamic responses (HDR) associated with cortical activation, fNIRS measures increase in total hemoglobin (THb) and oxygenated hemoglobin (OHb) levels while recording decreases in deoxygenated hemoglobin (DHb) concentrations in regions of interest (ROIs) (van de Rijt et al., 2016). Although fNIRS was introduced into clinical care nearly 40 years ago (Villringer et al., 1993; Chen et al., 2020), its application in studying brain development and NDDs has only recently gained attention (Vanderwert and Nelson, 2014). To date, fNIRS has primarily been used to investigate typical developmental processes such as speech perception and language, sensory and motor functions, social communication, and object and action processing in toddlers and children (Lloyd-Fox et al., 2010; Gervain et al., 2011). Its ability to capture brain activation from the earliest stages of development (Cabrera and Gervain, 2020) makes it particularly well-suited for studying NDDs.

This perspective paper explores the potential of fNIRS for analyzing visually-evoked hemodynamic responses (vHDR), assessing its viability as biomarker for brain function across both pediatric and adult populations.

## 2 The application of fNIRS in the study of the visual system

This section reviews key findings from studies employing fNIRS to analyze vHDR. The discussion is divided into two subsections: studies in adults and those in children. We summarize major findings, methodological approaches, and clinical applications while addressing the limitations of current research. Detailed technical information—including equipment, participant characteristics, and experimental paradigms—is systematically presented in Table 1 (adults) and Table 2 (children).

**Table 1:** The table summarizes fNIRS studies investigating visual processing in adults, presented chronologically to illustrate the evolving complexity of the visual paradigms utilized over time.

Reference	ROIs; channel count	Technical equipment	Wavelengths	Experimental paradigm	Sample characteristics	Mean age	Statistical analysis methods	Main findings
Villringer et al. (1993)	V1; N/A	NIRO-500, Hamamatsu	775, 825, 850, 904 nm	T-R design, flash-light stimulation	16 healthy subjects	27 y	N/A	fNIRS assesses brain activation via increased [OHb], decreased [DHb], independent of skin blood flow changes.
Meek et al. (1995)	V1; 1 ch	NIRO-500, Hamamatsu	779, 821, 855, 908 nm	T-R design, colored discs on grey background	10 healthy subjects	N/A	T-test and linear regression analysis	fNIRS measures visual stimulation effects on occipital cortex hemodynamics, showing regional [OHb] increases and blood volume changes.
Heekeren et al. (1997)	V1; N/A	NIRO-500, Hamamatsu	775, 825, 850, 904 nm	T-R design, multicolored dodecahedron	12 healthy subjects	29 y	T-test with Bonferroni correction and correlation analysis.	fNIRS reveals occipital hemodynamics during sustained visual stimulation: [OHb] rises, [DHb] fluctuates, highlighting temporal patterns critical for interpreting activation.
Heekeren et al. (1999)	V1; N/A	N/A	740–900 nm	T-R design, reversing checkerboard	10 healthy subjects	22.5 y	*T*-test and linear regression analysis	Visual stimulation increases hemoglobin oxygenation and oxidizes cytochrome-c oxidase.
Obrig et al. (2000)	V1; N/A	NIRO-500, Hamamatsu	775, 825, 850, 905 nm	T-R design, reversing checkerboard	5 healthy subjects	N/A	*T*-test and linear regression analysis	fNIRS detects low-frequency oscillations in cerebral oxygenation and metabolism.
Takahashi et al. (2000)	V1; 24 ch	Model N/A Hitachi Medical Co.	780, 830 nm	T-R design, checkerboard	5 healthy subjects	37 y	N/A	fNIRS monitors hemodynamic changes in the visual cortex during stimulation.
Colier et al. (2001)	V1; 2 ch	Custom device	775, 848, 901 nm	T-R design, pattern reversal checkerboard	6 healthy subjects	31.5 y	Wilcoxon signed-rank test	fNIRS effectively tracks occipital oxygenation bilaterally.
Gratton et al. (2001)	V1; 8 ch	Omnia® Tissue Oxymeter ISS Inc.	750 nm	T-R design, a frequency-varying colored grid	8 healthy subjects	23 y	*T*-test and linear regression analysis	Hemodynamic responses correlate linearly with neuronal activity, enabling quantitative neuroimaging of brain function.
Schroeter et al. (2004)	V1, V3, V5; 22 ch	ETG-100, Hitachi Medical Co.	780, 830 nm	T-R, checkerboard or array of red “L” shapes	9 healthy subjects	24.4 y	*T*-test, GLM and spectral analysis	fNIRS measures brain activation via hemoglobin changes; general linear models and spectral analysis enhance data reliability.
Plichta et al. (2006a)	Occipital and temporo-occipital cortices; 52 ch	ETG-4000, Hitachi Medical Co.	695, 830 ± 20 nm	T-R design, reversing checkerboard/ finger tapping task	18 healthy subjects	29 y	*T*-test, GLM, ICC, and correlation analysis	fNIRS detects regionally specific cortical activation, distinguishing occipital and motor task responses.
Plichta et al. (2006b)	Occipital cortex; 22 ch	ETG-4000, Hitachi Medical Co.	695, 830 ± 20 nm	T-R design, reversing checkerboard	12 healthy subjects	29 y	Paired *t*-test	The study investigated the retest reliability of event-related fNIRS. Results showed good group-level reliability,
Plichta et al. (2007)	Whole brain; 52 ch	ETG-4000, Hitachi Medical Co.	695, 830 ± 20 nm	T-R design, reversing checkerboard	15 healthy subjects	25.3 y	*T*-test, GLM and ANOVA	GLM-based analysis validates fNIRS for detecting visual cortex activation, confirming graded hemodynamic responses and enabling rapid event-related data interpretation.
Näsi et al. (2010)	V1; 16 ch	Custom device	N/A	T-R design, reversing checkerboard	10 healthy subjects	25.3 y	Correlation analysis and logarithmic regression	Hemodynamic responses (HDR) correlate linearly with visual-evoked potentials, outperforming stimulus duration in explaining HDR variance. Combined measurements enhance understanding of neuronal-hemodynamic interactions.
Rovati et al. (2007)	V1; N/A	NIMO, NIROX srl	685, 830 nm	T-R design, windmill pattern	9 healthy subjects	38.5 y	*T*-test, correlation and regression analysis	fNIRS and VEP correlate linearly, revealing visual contrast-dependent brain responses.
Kojima and Suzuki (2010)	Occipital cortex; 24 ch	ETG-4000, Hitachi Medical Co.	695, 830 nm	T-R design, pictures	25 healthy subjects	N/A	*T*-test and ANOVA	Active attention to visual scenes significantly increases OHb compared to passive viewing.
Minati et al. (2011)	V1; 4 ch	Oxymon Mk III, Artinis BV	764, 859 ± 20 nm	T-R design, reversing checkerboard	16 healthy subjects	25 y	*T*-test, ANOVA and linear regression analysis	Blood pressure fluctuations can confound brain NIRS, requiring continuous monitoring.
Wijeakumar et al. (2012)	Occipital cortex; N/A	N/A	690, 830 nm	T-R design, diverse checkerboards	10 healthy subjects	34 y	ANOVA	fNIRS revealed occipito-parietal activation to checkerboard stimuli, showing region-specific hemodynamic responses.
Thang et al. (2014)	V1; 7 ch	FOIRE-3,000, Shimadzu	780, 805, 830 nm	T-R design, flickering light	10 healthy subjects	20.6 y	*T*-test and ANOVA	fNIRS detects significant visual cortex response to flickering light stimuli.
Haigh et al. (2015)	V1 and prefrontal cortex; 10 ch	Oxymon Mk II Artinis Medical Systems	N/A	T-R design, diverse gratings	22 healthy adults	21.2 y	ANOVA and Pearson correlation	Stimulus parameters influence hemodynamic responses, reflecting cortical excitability dynamics.
Maggioni et al. (2015)	V1 and parieto-occipital cortex; 16 ch	DYNOT Compact, NIRx Medical Technologies	760, 830 nm	T-R design, reversing black and white screens	8 healthy subjects	28 y	GLM, t-test, Kruskal–Wallis test, and correlation analysis	Negative BOLD responses arise from reduced OHb and cerebral blood volume, explained via multimodal imaging integration.
Ward et al. (2015)	V1; 2 ch	OxiplexTS, ISS inc,	690, 839 nm	T-R design, reversing checkerboard	25 healthy subjects	Young: 20.5 y Old: 71.2 y	ANOVA, *t*-test, Pearson correlation	Healthy aging reduces the visual cortex’s hemodynamic response during visual stimuli.
Ward et al. (2016)	V1 and parieto-occipital cortex; 2 ch	OxiplexTS, ISS inc,	690, 830 nm	T-R design, random-dot stereograms	13 healthy subjects	24 y	ANOVA and *t*-test	fNIRS reveals parieto-occipital activation during depth perception via dynamic stereograms.
Cai et al. (2017)	V1 and parieto-occipital cortex; 8 ch	FOIRE-3000, Shimadzu Co.	780, 805, 830 nm	T-R design, random-dot stereograms	13 healthy subjects	24 y	GLM, ANOVA, *t*-test, and correlation analysis	fNIRS reveals parietal-occipital correlates of stereopsis, depth perception, and visual fatigue.
Maehara et al. (2007)	V1 and lateral occipital cortex; 24 ch	ETG-4000, Hitachi Medical Co.	695, 830 nm	T-R design, line drawings	30 healthy subjects	22.2 y	ANOVA	fNIRS reveals task-dependent differences in hemodynamic responses, highlighting specific cortical activations.
Zhao et al. (2019)	Occipital, parietal and temporal cortices; 44 ch	ETG-4000, Hitachi Medical Co.	695, 830 ± 20 nm	T-R design, visual search array	26 healthy subjects	22 y	GLM, ANOVA, *t*-test and correlation analysis	Cue-induced alpha and OHb lateralization predict neural attentional selection.
Pinti et al. (2021)	Occipital cortex; 16 ch	Custom device	780–900 nm	T-R design, reversing black-white and red-green checkerboards	13 healthy subjects	31 y	GLM (FIR), *t*-test, and cross-correlation analysis	fNIRS, combined with EEG, enhances neuroimaging by linking brain metabolism and haemodynamics to neuronal activity.
Mazziotti et al. (2022)	Occipital cortex; 22 ch	NIRSPort2, Nirx Medical Technologies	760, 850 nm	T-R design, cartoon-based checkerboard	59 healthy subjects (40 adults and 19 children)	Adults: 31y Children: 7y	*T*-test, ANOVA and correlation analysis	fNIRS measures hemodynamic responses in the occipital cortex, correlating with autistic traits. A cartoon-like, entertaining procedure enhances compliance for children.
Miki et al. (2005)	Occipital cortex; N/A	OM-100A, Shimadzu Co.	780, 805, 830 nm	T-R design, flash-light stimulation	6 healthy subjects and 5 patients with unilateral optic neuritis	Controls: 32 y Patients: 34.5 y	*T*-test	NIRS detects reduced occipital activation in optic neuritis during visual stimulation.
Coutts et al. (2012)	V1; 8 ch	Artinis Medical Systems, model N/A	N/A	T-R design, checkerboard or gratings	20 healthy subjects and 20 patients with migraine	44 y	ANOVA, *t*-test and cross-correlation analysis	Migraine patients show altered hemodynamic response to visual stimuli, improved with ophthalmic filters.
Re et al. (2021)	V1; 2 ch	Custom device	687, 826 nm	T-R design, reversing checkerboard	31 healthy subjects, 43 patients with glaucoma and12 mixed subjects	67 y	Logistic regression and ROC analysis	Glaucomatous eyes show altered hemodynamic responses, distinguishing them from healthy eyes.
Zhang Y. et al. (2022)	Occipital cortex; 22 ch	ETG-4000, Hitachi Medical Co.	695, 830 nm	T-R design, reversing checkerboard	19 healthy subjects and 22 myopic adults	22 y	GLM, *t*-test and repeated measures ANOVA	Myopic defocus decreases visual cortex oxygenation, reversible by optical correction.
de Tommaso et al. (2022)	Parieto-occipital cortex; 16 ch	NIRSport, Nirx Medical Technologies	760, 850 nm	T-R design, reversing checkerboard	10 healthy subjects and 13 patients with migraine	Controls: 48.8 y Patients: 47 y	GLM, *t*-test and repeated measures ANOVA	Galcanezumab treatment reduces visual cortex reactivity in migraine patients, indicating potential disease-modifying effects.

Papers focused on pathological conditions are marked in bold. Key features include technical information, including visual regions activated (e.g., V1: primary visual cortex; V3: third visual complex; V5: middle temporal visual area), fNIRS device, channel count, wavelengths used, experimental design (T-R design: task-related), and participant demographics (e.g., age in years, y). Hemodynamic parameters measured include [OHb] (oxygenated hemoglobin) and [DHb] (deoxygenated hemoglobin), with spatial data captured via channels (ch). Statistical analysis and main findings raised are also listed. SD: standard deviation; GLM: General Linear Model; ICC: IntraClass Correlation; FIR: finite impulse response; ROC: Receiver Operating Characteristic. Entries with unavailable information are noted as N/A.

**Table 2:** The table summarizes fNIRS studies investigating visual processing in children.

Reference	ROIs; channel count	Technical equipment	Wavelengths	Experimental paradigm	Sample characteristics	Age range	Statistical analysis methods	Main findings
Meek et al. (1998)	Occipital cortex and frontoparietal region; N/A	NIRO500, Hamamatsu Photonics	780, 810, 850 ± 5 nm	T-R design, reversing checkerboard	20 healthy subjects	3 d-14 wk	*T*-test	Visual stimulation increases hemoglobin levels in infants’ visual cortex.
Taga et al. (2003a)	Occipital and frontal cortex; 24 ch	NIR OT, Hitachi Medical Co.	780, 830 nm	T-R design, reversing checkerboard	7 healthy subjects	2–4 m	ANOVA	Evidence of vHDR in infants aged 2–4 months in localized areas of the occipital cortex, no more detectable in the frontal cortex.
Taga et al. (2003b)	Occipital and frontal cortex; 24 ch	NIR OT, Hitachi Medical Co.	780, 830 nm	T-R design, flashing lights	16 healthy subjects	1–9 d	ANOVA	Near-infrared optical topography reveals newborn visual and prefrontal cortex activation.
Kusaka et al. (2004)	Occipital cortex; 24 ch	OMM-2000, Shimadzu Corp.	776, 804, 828 nm	T-R design, flashing lights	10 healthy subjects (5 infants and 5 adults)	Children: 29–111 d Adults: N/A	Wilcoxon signed-rank test and Mann–Whitney U test	Infant and adult visual cortex responses differ due to developmental changes.
Wilcox et al. (2005)	V1 and inferior temporal cortex; 8 ch	Custom device	690, 830 nm	T-R design, object processing task	7 healthy subjects	6.5 m	N/A	fNIRS shows neural activation in infants’ visual and temporal cortices during object processing.
Watanabe et al. (2008)	Occipital and frontal cortex; 48 ch	ETG-100, Hitachi Medical Co.	780, 830 nm	T-R design, colorful mobile objects and reversing checkerboard	35 healthy subjects	93–125 d	*T*-test with FDR correction	Infant cortical regions functionally dissociate, activating differently for visual stimuli.
Karen et al. (2008)	Occipital cortex; 10 ch	Custom device	730, 830 nm	T-R design, flashing red lights	15 healthy subjects	2–9 d	Paired Wilcoxon signed-rank test	fNIRS detects brain oxygenation changes in newborns, resembling adult responses.
Wilcox et al. (2008)	V1 and inferior temporal cortex; N/A	Custom device	690, 830 nm	T-R design, object processing task	35 healthy subjects	6.5 m	*T*-test and ANOVA	fNIRS detects brain responses to object processing in infants’ visual cortices.
Liao et al. (2010)	Occipital and motor cortex; 26 ch	Custom device	750, 850 nm	T-R design, reversing checkerboard	10 healthy subjects	1–3 d	*T*-test	High-density fNIRS improves brain activation signals in infants, detecting adult-like neurovascular coupling.
Watanabe et al. (2010)	Occipital and frontal cortex; 48 ch	ETG-100, Hitachi Medical Co.	780, 830 nm	T-R design, colorful mobile objects and reversing checkerboard	40 healthy subjects	74–89 d	*T*-test with FDR correction, GLM	This study suggests functional cortical regions for visual perception emerge between 2–3 months.
Remijn et al. (2011)	V1 and temporo-parietal cortex; 37 ch	Foire 3,000, Shimadzu	780, 805, 830 nm	T-R design, Japanese TV series (static and motion epochs)	45 healthy subjects (26 infants and 19 adults)	Infants: 3–4 y Adults: 20–41 y	*T*-test and ANOVA	Preschool children show greater OHb response than adults to visual stimuli.
Watanabe et al. (2012)	Whole brain; 94 ch	ETG-7000, Hitachi Medical Co.	785, 830 nm	T-R design, high-luminance unpatterned stimuli and reversing checkerboard	35 healthy subjects	6 m	*T*-test and GLM with FDR correction	High-luminance stimuli evoke occipital deactivation and prefrontal activation in infants.
Watanabe et al. (2013)	Occipital, temporal, and frontal cortices; 96 ch	ETG-7000; Hitachi Medical Co.	785, 830 nm	T-R design, colorful mobile objects	50 healthy subjects	101–123 d	*T*-test with FDR correction	The study shows multisensory processing in infants involves region-specific cortical activation.
Emberson et al. (2015)	Occipital and temporal cortices; 24 ch	ETG-400, Hitachi Medical Co.	N/A	T-R design, combination of visual and auditory stimuli	37 healthy subjects	5–7 m	*T*-test	Infants’ occipital cortex exhibits expectation-based feedback during sensory processing, modulating responses.
Emberson et al. (2017)	Occipital and temporal cortices; 24 ch	ETG-400, Hitachi Medical Co.	N/A	T-R design, object recognition tasks	32 healthy subjects	6 m	*T*-test and ANOVA	fNIRS reveals early functional specialization of the lateral occipital cortex in infants.
Hirshkowitz et al. (2018)	Occipital, parietal and temporal cortices; 12 ch	TechEn, Inc. model N/A	690, 830 nm	T-R design, white dots against a black background	14 healthy subjects	4–6 m	*T*-test and Bayesian analysis	Infants use motion to perceive 3-D shapes, activating specific cortical regions.
Werchan et al. (2018)	Occipital and temporal cortices; 8 ch	CW6 system, TechEn Inc.	690, 830 nm	T-R design, red or green ball on a rectangular grid of white dots and black background	65 healthy subjects	3.5–5 m	ANOVA and correlation analysis	Infant occipital and temporal cortices are functionally coupled during multisensory processing.
Boldin et al. (2018)	Occipital, temporal, and frontal cortices; 24 ch	ETG-400, Hitachi Medical Co.	695, 830 nm	T-R design, combination of visual and auditory stimuli	79 healthy subjects (36 full-term and 43 preterm infants)	5–7 m	Linear and quadratic mixed-effects models; multiple regression analysis	Prematurity disrupts sensory processing during learning, affecting occipital cortex modulation.

Key features include technical information, including visual regions activated (e.g., V1: primary visual cortex), fNIRS device, channel count, wavelengths used, experimental design (T-R design: task-related), and participant demographics [e.g., age in years (y), months (m), weeks (wk) or days (d)]. Spatial data were captured via channels (ch). We chose to present the age range as it provides a more comprehensive and meaningful context for understanding developmental variability in infants younger than 12 months compared to a single average age. Preterm infants' age was corrected based on their gestational age. Statistical analysis and main findings raised are also listed. FDR: False Discovery Rate; GLM: General Linear Model. Entries with unavailable information are noted as N/A.

### 2.1 Visual cortical processing through fNIRS: methodological insights and clinical applications in adults

Early studies in the 1990s used the visual system as a model to investigate cortical HDR during task-related experiments in healthy adults. Villringer’s and Zeki’s group employed various visual stimuli- including flashes, pictures, and moving colored shapes- to examine occipital cortex activation. These studies revealed a distinct HDR pattern, with increased OHb levels and a smaller-magnitude decrease in DHb, peaking approximately 5 s after stimulus onset. This pattern underscored fNIRS’s potential as a bedside tool for monitoring brain activity with minimal interference from superficial blood flow (Villringer et al., 1993; Meek et al., 1995). Subsequent research explored the temporal dynamics of vHDR during sustained stimulation, showing that OHb levels rise and remain elevated, while DHb initially decreases before returning to baseline and exhibiting a post-stimulus overshoot (Heekeren et al., 1997). Multi-channel fNIRS studies confirmed the regional specificity of these responses, with activation localized to the contralateral occipital cortex reinforcing the stimulus-specific nature of vHDR (Colier et al., 2001; Plichta et al., 2006a; Wijeakumar et al., 2012).

Over the years, experimental paradigms have refined visual stimulation parameters- such as shape, contrast, chromaticity, and frequency- to optimize vHDR reliability (Gratton et al., 2001; Rovati et al., 2007; Wijeakumar et al., 2012; Thang et al., 2014; Haigh et al., 2015). High-contrast black-and-white checkerboard and pattern-reversal gratings have proven the most effective and are now widely used in fNIRS studies of visual processing (Heekeren et al., 1999; Obrig et al., 2000; Takahashi et al., 2000; Wijeakumar et al., 2012; Haigh et al., 2015). Higher stimulus frequencies produce linear increases in vHDR amplitude, whereas greater contrast elicits logarithmic changes in OHb and DHb levels (Gratton et al., 2001; Rovati et al., 2007). These findings highlight how optimizing visual stimulation parameters can enhance the reliability and robustness of fNIRS measurements in capturing brain activity.

Recent studies have expanded beyond primary visual processing to examine associative pathways in extrastriate cortices. Tasks involving depth perception, selective attention, and shape recognition have provided insights into the broader visual networks and their roles in higher-order cognitive functions (Ward et al., 2016; Cai et al., 2017; Maehara et al., 2007; Zhao et al., 2019). Advanced analytical methods, including general linear models and spatially resolved spectral techniques, have further enhanced fNIRS capability to detect cortical activity (Schroeter et al., 2004; Plichta et al., 2007). Additionally, multi-modal approaches integrating fNIRS with EEG or fMRI have validated fNIRS’s reliability by demonstrating strong signal consistency across modalities (Rovati et al., 2007; Näsi et al., 2010; Maggioni et al., 2015; Chen et al., 2017; Zhao et al., 2019; Pinti et al., 2021).

Building from the data collected in young adults, studies on healthy aging have revealed increased variability in vHDR, with OHb and DHb responses becoming less distinct between stimulation and rest in older adults. This pattern likely reflects age-related declines in neural efficiency and vascular responsiveness (Ward et al., 2015).

In clinical settings, fNIRS has been applied to ophthalmological conditions such as optic neuritis, myopia, and glaucoma, identifying distinct HDR alterations as potential biomarkers of visual impairment (Miki et al., 2005; Zhang Y. et al., 2022; Re et al., 2021). For example, optic neuritis patients exhibit reduced occipital HDR during affected-eye stimulation, despite normal visual acuity recovery, suggesting persistent neural deficits (Miki et al., 2005). Additionally, variations in vHDR patterns have been associated with disease severity in glaucoma and myopia, underscoring the diagnostic potential of fNIRS (Zhang Y. et al., 2022; Re et al., 2021).

The fNIRS has also offered valuable insights into neurological disorders. Studies in migraine patients have shown increased OHb levels during visual stimulation, which normalized after treatment with Galcanezumab, correlating with symptom relief (Coutts et al., 2012; de Tommaso et al., 2022). These findings highlight the potential of fNIRS in monitoring treatment efficacy and investigating changes in visual processing across a range of neurological conditions.

### 2.2 fNIRS for assessing visual processing and cortical development in infants and children

The functional Near Infrared Spectroscopy has become a key tool for investigating brain activity and development in infants and children, offering insights into functional specialization, cortical maturation, and sensory processing (Lloyd-Fox et al., 2010; Gervain et al., 2011).

Hemodynamic responses to visual stimuli have been observed from the earliest stages of life. Studies using checkerboard patterns in awake infants aged 3 days to 16 weeks have demonstrated regionally specific OHb and DHb changes (Meek et al., 1998; Taga et al., 2003a; Liao et al., 2010). These findings highlight fNIRS’s high spatial specificity, even in very young subjects, and suggest the presence of early cerebrovascular coupling that closely resembles adult patterns.

However, interpreting infant visual fNIRS data requires careful consideration. Unlike adults, neonates often exhibit increased DHb levels during stimulation likely due to immature neurovascular coupling, which typically stabilizes by 3–4 years of age (Meek et al., 1998; Kusaka et al., 2004). Additionally, factors such as consciousness state and stimulus characteristics influence HDR reliability. Checkerboard patterns consistently evoke robust responses, whereas less structured stimuli yield weaker activations (Watanabe et al., 2012). Furthermore, early sensory processing in neonates involves prefrontal cortical contributions, with neural circuits progressively maturing into an adult-like configuration over time (Taga et al., 2003a,b; Karen et al., 2008).

Building on these foundational studies, research has explored functional differentiation within visual cortical circuits. Studies in 3-month-old infants revealed that primary visual regions respond similarly to both object (e.g., moving objects) and non-object (e.g., checkerboard) stimuli, whereas the lateral occipital cortex selectively activates in response to object stimuli. The same pattern is replicated in preschool children (Watanabe et al., 2008; Remijn et al., 2011; Hirshkowitz et al., 2018). By 5–6 months of age, the inferior temporal and the lateral occipital cortices support feature-based object recognition, with infants distinguishing objects by shape and size before incorporating color clues (Wilcox, 1999; Wilcox et al., 2005; Wilcox et al., 2008; Emberson et al., 2017). These findings further indicate that stable functional segregation within the visual cortex is present from an early age. Longitudinal studies further indicate that functional specialization follows a developmental trajectory, shifting from broad, undifferentiated cortical activation at 2 months to region-specific processing by 3 months of age (Watanabe et al., 2010).

Recent research has also explored the emergence of multisensory integration in infants. Audiovisual stimuli enhance activation in both sensory-specific areas and strengthen functional connectivity between the occipital and temporal cortices, emphasizing the role of early multisensory experience in shaping neuronal networks (Watanabe et al., 2013; Emberson et al., 2015; Werchan et al., 2018 but see also Bortfeld et al., 2007).

Beyond typical development, fNIRS has been valuable for studying high-risk populations, such as preterm infants. Although research on pathological visual processing using fNIRS are limited, recent studies suggest that prematurity may disrupt functional connectivity and top-down modulation, leading to inefficient information transfer and potential learning difficulties (Boldin et al., 2018). These findings highlight fNIRS potential for early cerebral dysfunction detection, enabling timely interventions to mitigate developmental challenges.

## 3 Discussion

This perspective paper provides a comprehensive review of visual paradigms employed in fNIRS studies across adult and pediatric populations, emphasizing its potential as a biomarker for monitoring brain function. By capturing vHDR, fNIRS emerges as a promising tool in diagnosing and tracking NDDs and assessing treatment responses, advancing the field of personalized medicine.

Seminal studies have established that fNIRS reliably detects task-specific cortical activation across ages, with checkerboard stimuli consistently eliciting robust and reproducible vHDR in both adults and children (Villringer et al., 1993; Meek et al., 1995; Meek et al., 1998; Taga et al., 2003a). These foundational findings set the stage for its use in mapping developmental trajectories and revealing neural alterations in both research and clinical settings. More recent studies have applied fNIRS to pathological conditions such as migraines, visual impairments, and prematurity (Miki et al., 2005; Boldin et al., 2018; Re et al., 2021; Zhang Y. et al., 2022a; de Tommaso et al., 2022), demonstrating its ability to distinguish pathological states, predict disease severity, and monitor treatment efficacy. For example, studies in glaucoma and migraine patients have shown that fNIRS can detect disease-specific hemodynamic alterations and track therapeutic responses (Re et al., 2021; de Tommaso et al., 2022). Additionally, research on prematurity has highlighted how early disruptions in neural connectivity affect sensory processing and cognitive development, reinforcing fNIRS’s potential in detecting and addressing developmental vulnerabilities (Boldin et al., 2018).

In the study of NDDs, fNIRS has gained traction as a tool for identifying divergent hemodynamic patterns in conditions such as Attention Deficit Hyperactivity Disorder (ADHD), Autism Spectrum Disorder (ASD), and Developmental Coordination Disorder (DCD) (Conti et al., 2022; Scaffei et al., 2023; Su et al., 2023; Huang et al., 2024; Kurane et al., 2024). Resting-state and longitudinal connectivity studies have revealed abnormalities in the temporal dynamics of brain activity, shedding insights on developmental deviations in children with NDDs (e.g., Keehn et al., 2013; Cao et al., 2021; Zhang F. et al., 2022). Event-related studies have further identified distinct alterations in HDR, such as atypical prefrontal cortex activation in ADHD (Ishii et al., 2017; Yasumura et al., 2019) and reduced vHDR amplitude in preschool females with high-functioning ASD (Scaffei et al., 2023), reinforcing the potential of task-related fNIRS as a non-invasive and accessible diagnostic and monitoring tool. However, the methodological variability in data collection and analysis presents significant challenges, limiting the comparability of results across studies and hindering the broader clinical application of fNIRS. To overcome these limitations, there is an urgent need for standardized protocols that encompass every stage of the experimental process. These protocols should provide clear guidelines for stimulation paradigms, as well as establish specific requirements for fNIRS equipment, including wavelength, source-detector distance, and channel configuration. In addition, robust signal processing procedures must be defined, with a particular focus on motion artifact correction techniques, such as short-channel regression or independent component analysis, along with appropriate filtering and data analysis methods. Finally, implementing rigorous reporting standards is essential to guarantee transparency, reproducibility, and the ability to effectively compare findings across studies.

For X-linked NDDs, preclinical research has demonstrated that visually evoked hemodynamic changes can distinguish between healthy and pathological conditions (Mazziotti et al., 2017; Mazziotti et al., 2020). These findings suggest that vHDR could serve as a non-invasive tool for early diagnosis and disease monitoring in clinical populations. However, it is important to note that the current evidence is primarily derived from animal models. Therefore, future research should prioritize examining vHDR in human cohorts with X-linked NDD to validate the translational potential of fNIRS. Additionally, to fully realize the potential of visual fNIRS as a functional biomarker, it is essential to broaden its application across a wide range of NDDs. Investigating fNIRS biomarkers in various NDDs—encompassing genetic, idiopathic, and environmentally influenced disorders—will deepen our understanding of both shared and disorder-specific mechanisms. This expanded focus will ultimately enhance diagnostic precision and support the development of more personalized intervention strategies.

Despite its promises, several critical factors influence the reliability and clinical utility of fNIRS as a biomarker, including its restricted brain penetration, feasibility, test–retest reliability, age-related variations in response amplitudes, and engagement-dependent fluctuations in hemodynamic responses. fNIRS has proven to be highly feasible even in challenging populations, including infants and children with NDDs (Vanderwert and Nelson, 2014). However, participant engagement significantly impacts the quality of vHDR recordings, as active tasks tend to produce stronger responses compared to passive viewing (Kojima and Suzuki, 2010). To improve compliance, particularly in young children, innovative protocols combining animated stimuli with traditional checkerboard paradigms have been developed, enhancing both engagement and data quality (Mazziotti et al., 2022).

Age-related anatomical differences, such as thinner skulls and scalps in children, influence light propagation during fNIRS measurements, highlighting the need for age-stratified interpretations of biomarkers (Remijn et al., 2011; Mazziotti et al., 2022). Additionally, while short-term reproducibility of visual event-related hemodynamic responses has been demonstrated in adults (Plichta et al., 2006b), further research is required to assess the long-term stability and circadian effects on fNIRS measurements. Moreover, controlling for systemic physiological factors, such as blood pressure and heart rate fluctuations, is essential to improve the precision of fNIRS-based biomarkers (Minati et al., 2011).

In conclusion, fNIRS, particularly in visual paradigms, is a powerful, non-invasive tool for assessing brain function, complementing existing technologies such as fMRI and EEG, in both typical and atypical development. Expanding standardized protocols and refining vHDR analysis will strengthen its clinical applications, enabling earlier and more accurate diagnoses, tracking neurodevelopmental trajectories, and optimizing therapeutic strategies. Given the urgent need for reliable functional biomarkers in X-linked NDDs, expanding fNIRS studies across a broader spectrum of conditions will be essential. Furthermore, integrating fNIRS into a multimodal framework, such as combining it with EEG, could provide transformative insights into brain function. EEG offers high temporal resolution complementing fNIRS’s spatial information, thus enabling a more comprehensive assessment of neural activity. The potential of multimodal imaging is also evident in recent studies that simultaneously collect fNIRS and fMRI data to investigate the relationship between cortical hemodynamics and deeper brain activity. For example, the feasibility of using fMRI-measured cortical activity has been demonstrated to predict deep-brain activity, showing a strong correlation with fNIRS measurements. Since many deep brain regions are anatomically and functionally connected with cortical surface regions (Bullmore and Sporns, 2009), this suggests that fNIRS, when combined with fMRI-informed models, can extend its utility beyond its typical limitation of superficial cortical sensitivity (Liu et al., 2015; Balters et al., 2023).

Future research should also focus on validating fNIRS measures, such as vHDR, against established clinical endpoints, including cognitive assessments (e.g., Bayley Scales of Infant Development, Wechsler Intelligence Scale for Children, Vineland Adaptive Behavior Scale, etc.) and motor function scales (e.g., Gross Motor Function Measure). This validation is essential for confirming the clinical relevance of fNIRS biomarkers and for establishing their ability to predict disease severity and treatment outcomes.

## Data Availability

The original contributions presented in the study are included in the article/supplementary material, further inquiries can be directed to the corresponding author.
